# A breakdown in communication? Understanding the effects of aging on the human small intestine epithelium

**DOI:** 10.1042/CS20150364

**Published:** 2015-07-03

**Authors:** Neil A. Mabbott

**Affiliations:** * The Roslin Institute & Royal (Dick) School of Veterinary Sciences, University of Edinburgh, Easter Bush, Midlothian EH25 9RG, U.K.

**Keywords:** aging, gut permeability, immunosenescence, interleukin-6, intestine

## Abstract

In the intestine, a single layer of epithelial cells sealed together at their apical surfaces by tight junctions helps to prevent the luminal commensal and pathogenic micro-organisms and their toxins from entering host tissues. The intestinal epithelium also helps to maintain homoeostasis in the mucosal immune system by expressing anti-inflammatory cytokines in the steady state and inflammatory cytokines in response to pathogens. Although the function of the mucosal immune system is impaired in elderly humans, the molecular mechanisms which cause this dramatic functional decline are poorly understood. Our current understanding of the effects of aging on the physical and immunological properties of the intestinal epithelial barrier is also very limited. In this issue of *Clinical Science*, Man et al. provide further insight into the effects of aging on small intestinal barrier function in humans and the influence that gut luminal micro-organisms may have on it. Using human terminal ileal biopsy tissues they show that intestinal permeability to solutes, but not macromolecules, was significantly increased in the intestines of elderly humans. This was accompanied by elevated expression of the pro-inflammatory cytokine interleukin (IL)-6 which appeared to modulate claudin-2 expression and solute permeability in the epithelium. Conversely, IL-8 synthesis in response to flagellin stimulation was reduced in intestines of the elderly subjects, but was not associated with effects on Toll-like receptor 5 (TLR5) expression. These data provide an important advance in our understanding on the effects of aging on intestinal permeability and innate mucosal immune responsiveness in elderly humans.

## Abstract (precis)

A new study by Man and colleagues provides further insight into the effects of aging on small intestinal barrier function in humans. Here, their findings are briefly summarised and the wider implications discussed.

The mammalian intestine contains a huge burden of commensal micro-organisms which in the lumen of the human colon are estimated to be as high as 3×10^11^/g of contents. These commensal micro-organisms are prevented from entering host tissues by a single layer of epithelial cells (enterocytes) sealed together at their apical surfaces by tight junctions. Tight junctions comprise complexes of transcellular proteins, including occludins and claudins, which bind together the plasma membranes of adjacent enterocytes and play a central role in regulating mucosal permeability. An impermeable barrier is formed which prevents the pericellular leakage of luminal solutes and particles such as micro-organisms and their toxins, digestive enzymes and undigested food molecules into the underlying tissue. The mucosal immune system of the gastrointestinal tract provides an important first line of defence against orally acquired pathogens. As well as detecting and destroying pathogens and their toxins, the mucosal immune system must also be able to discriminate between these pathogens and the harmless commensal micro-organisms and food antigens and generate tolerance towards them. The intestinal epithelium plays a significant role in homoeostasis of the mucosal immune system by the expression of anti-inflammatory cytokines in the steady state and inflammatory cytokines in response to certain pathogenic stimuli.

The function of the mucosal immune system is impaired in elderly humans [1]. This aging-related decline in immune function is termed immunosenescence and is associated with a decreased capacity to induce protective immunity and generate tolerance towards harmless antigens, suboptimal vaccine efficacy and the increased incidence of tumours and autoimmune diseases. As a consequence, the incidence of gastrointestinal pathogen infections is higher in elderly humans and is a major cause of morbidity and mortality.

The precise cellular and molecular mechanisms which cause the dramatic functional decline in the mucosal immune system are poorly understood. A common feature in elderly individuals is a low-grade background chronic inflammation, termed inflammaging [2], which results from an imbalanced expression of anti-inflammatory and inflammatory cytokines. This aberrant inflammation is considered to impair immunity to pathogens.

It is generally perceived that the intestinal barrier becomes leaky as we age, developing the so-called ‘leaky gut’. However, our current understanding of the effects of aging on the physical and immunological properties of this important epithelial barrier is very limited and often conflicting. Interactions between the intestinal epithelium and the intestinal microbiota are considered to play an important role in maintaining the low-grade chronic inflammation in the elderly [3]. Therefore, in this issue of *Clinical Science*, Man et al. [4] provide further insight into the effects of aging on the small intestinal barrier function in humans and the influence that gut luminal micro-organisms may have on it.

In their study, intestinal barrier characteristics and immunological properties were compared in terminal ileal biopsy tissues from healthy aged (66–77 years of age), young (7–12 years of age) and adult (20–40 years of age) humans. *Ex vivo* analyses suggested that intestinal permeability to solutes was significantly increased in the intestines of elderly humans, as assessed by measuring transepithelial electrical resistance (TEER, indicative of the ionic gradient) across the mucosal epithelia of freshly collected ileal biopsies. However, this apparent increased leakiness was not due to disturbances to the overall morphology of the tight-junctions. Permeability to macromolecular particles, in contrast, was not affected by aging.

In the steady state, the gut epithelium secretes certain anti-inflammatory cytokines which help to regulate intestinal homoeostasis and the host response to the intestinal microbiota, whereas the presence of pathogens can induce the expression of inflammatory cytokines to stimulate an immune response against them. Some pro-inflammatory cytokines such as interleukin (IL)-1β, IL-6, interferon (IFN)-γ and tumour necrosis factor (TNF)-α may also influence mucosal barrier integrity and tight junction status. A series of *ex vivo* and *in vitro* experiments were therefore undertaken to determine whether the cytokine profile was disturbed in the elderly intestine in the steady state, and if so, whether it might also influence intestinal permeability. Expression of the pro-inflammatory cytokine IL-6 was shown to be significantly elevated in elderly intestines when compared to those from younger individuals. Many cell populations within the intestine may express IL-6, but in the study by Man et al. [4] CD11c^+^CD45^+^ cells (mononuclear phagocytes) appeared to represent a major source. The precise nature of the CD11c^+^ cells which express high levels of IL-6 in the aged intestine was not determined. Mononuclear phagocytes are a heterogeneous population of macrophages and dendritic cells and the specific depletion of CD11c^+^ cells in the mouse ablates both populations in the small intestine [5].

IL-6 has been proposed to play an important role in the regulation of tight junctions in the intestinal epithelium where it stimulates the expression of claudin-2, which is associated with increased tight junction permeability [6]. Although expression of the important tight junction components zonula occuldens protein (ZO)-1, occludin and junctional adhesion molecule 1 (JAMA-1) was unaffected in the intestines of elderly individuals, claudin-2 expression was increased. Whether mononuclear phagocyte-derived IL-6 mediates the increased permeability of the aging intestinal epithelium to solutes *in vivo* remains to be determined. However, conditioned medium from cultivated aged intestinal biopsies induced a significant decrease in TEER when applied to a monolayer of human colonic epithelial cells (Caco2 cells) and was accompanied by an increase claudin-2 expression. Importantly, in the presence of neutralizing anti-IL-6 antibodies, the reduction in epithelial permeability and up-regulated expression of claudin-2 were prevented. These observations raise many important questions, including the following. What is the initial factor which induces IL-6 expression in the elderly intestine? Will modulation of IL-6 expression provide a novel method reduce solute permeability in the aging human intestine and in doings so improve immune responsiveness? Throughout their study, biopsies of terminal ileum were analysed. Thus whether the effects described are specific to this anatomical site, or are similar in other sections of the intestine also remains to be determined.

Man et al. [4] then went on to determine whether host responses to bacteria-derived components were dysregulated in the elderly. Bacterial flagellin is a pathogen-associated molecular pattern which can be recognized on host cells via surface expression of Toll-like receptor 5 (TLR5) to initiate a protective immune response. Stimulation of terminal ileal biopsies with bacterial flagellin induced significant expression of the inflammatory cytokine IL-8 in tissues from young individuals, but this response was much reduced in those from elderly individuals. However, host age did not influence TNFα synthesis after treatment with a live probiotic mixture. The molecular mechanism responsible for the aging-related decline in IL-8 expression in the aged intestine is uncertain since TLR5 expression was not affected. The effects observed may plausibly be due to reduced expression of important TLR5 intracellular signalling molecules, such as myeloid differentiation primary response protein (MyD88) or TIR-domain-containing adapter-inducing interferon-β (TRIF) or alternatively due to reduced expression of the intracellular NOD-like receptor protein 4 (NLRC4) which can recognize peptidoglycan in the cytosol. Epigenetic factors can also modulate the induction of IL-8 expression in intestinal epithelial cells [7]. Since epigenetic factors such as DNA methylation and histone modification are influenced by aging, this suggests an alternative mechanism by which aging may affect the immune response in the intestinal epithelium.

Interactions between the immune system and the gut microbiota can influence the development of the mucosal immune system, which can in turn influence the development of the microbiota. For example, the production of short-chain fatty acids such as butyrate by the microbial fermentation of dietary fibre can help to regulate inflammatory responses in the intestine [8]. Changes to the diversity of the gut microflora in and elderly individuals correlate strongly with effects on health status and may contribute towards their increased inflammatory status [9]. Whether the aging-related changes to the microbiota trigger the changes to the permeability of the intestinal epithelium remains to be determined, but it is interesting to note that elevated systemic levels of IL-6 and IL-8 were accompanied by significant changes to the gut microbiota and a reduction in butyrate-producing bacteria in elderly individuals [9].

Man et al. [4] provide an important account of the influence of aging on intestinal permeability and the innate mucosal immune responsiveness in elderly humans. To date, the literature on the effects of aging on intestinal permeability has been contradictory. As always ‘the devil is in the detail’ and factors such as the host species, anatomical site and the method used to determine gut permeability may each have a significant influence [10]. Therefore, selection of the most appropriate physiologically relevant model system in which to study the effects of aging on the intestinal epithelial barrier is critical to significantly advance the progress in our current understanding. Identification of the cellular and molecular factors which affect intestinal permeability or trigger inflammaging may identify novel methods to restore gut integrity and improve mucosal immune responsiveness in elderly humans.

## Abbreviations

IFN: interferon
IL: interleukin
TEER: transepithelial electrical resistance
TLR5: Toll-like receptor 5
TNF: tumour necrosis factor
ZO-1: zona occludens protein 1
